# Teclistamab for multiple pulmonary lesions in extramedullary myeloma: first successful case and review of the literature

**DOI:** 10.1007/s00277-026-07011-2

**Published:** 2026-04-25

**Authors:** Teruhito Takakuwa, Kensuke Ohta, Yusuke Tanada, Yusuke Okayama, Masayuki Hino

**Affiliations:** 1 Department of Hematology, Wakakusa-Daiichi Hospital, 1-6, wakakusa-cho, Higashi-osaka, Osaka 579-8056 Japan; 2 Hematology Ohta Clinic, Shinsaibashi, Osaka Japan

**Keywords:** Teclistamab, Relapsed/refractory multiple myeloma, Extramedullary disease, Pulmonary involvement, Bispecific antibody

## Abstract

Extramedullary disease (EMD) in multiple myeloma (MM) is associated with aggressive clinical behavior and poor prognosis, particularly when involving the lung parenchyma, which is an extremely rare manifestation. Bispecific antibodies targeting B-cell maturation antigen (BCMA), such as teclistamab, have recently demonstrated promising efficacy in heavily pretreated relapsed/refractory multiple myeloma (RRMM). However, evidence regarding their activity in patients with pulmonary extramedullary involvement remains limited. We report the case of a 76-year-old man with IgA-κ RRMM who developed multiple extramedullary lesions, including pulmonary nodules, after progression on several lines of therapy. Following initiation of teclistamab, the patient experienced grade 1 cytokine release syndrome that resolved rapidly after tocilizumab administration. An abdominal wall mass transiently enlarged early after treatment initiation, raising suspicion of pseudoprogression. Subsequently, all extramedullary lesions, including the pulmonary nodules, showed marked regression, with complete radiologic resolution observed by day 80. At 4.5 months after treatment initiation, both positron emission tomography–computed tomography and bone marrow multiparameter flow cytometry demonstrated minimal residual disease negativity. The patient has continued teclistamab therapy with sustained disease control. This case illustrates that teclistamab may provide meaningful clinical benefit even in RRMM with pulmonary extramedullary involvement, a condition typically associated with extremely poor outcomes. Our findings also suggest that transient lesion enlargement early after therapy may represent pseudoprogression and should be interpreted cautiously in patients receiving bispecific antibody therapy.

## Introduction

Multiple myeloma (MM) patients who are triple-class exposed and/or refractory to immunomodulatory agents (IMiDs), proteasome inhibitors (PIs), and anti-CD38 monoclonal antibodies are known to have extremely poor outcomes, with a median progression-free survival of approximately 4.6 months and a median overall survival of only 9–14 months [1–3]. In recent years, chimeric antigen receptor T-cell therapy and bispecific antibodies (BsAbs) have emerged as promising therapeutic options for patients with triple-class–exposed relapsed/refractory multiple myeloma (RRMM). In the MajesTEC-1 study, teclistamab has demonstrated remarkable efficacy even in heavily pretreated populations, with overall response rates (ORR) of 52.5% and 30.0% of patients achieving complete response (CR) or better [4]. Consistent depth of response and manageable safety profiles have also been confirmed in Japanese clinical settings [5].

A large multicenter retrospective study led by the International Myeloma Working Group Immunotherapy Committee evaluated 210 patients treated across nine academic centers in five countries. Notably, 71% of these patients would have been considered ineligible for the MajesTEC-1 trial due to prior BCMA-targeted therapy, severe renal dysfunction, profound cytopenias, or poor performance status. Despite this highly challenging clinical background, the ORR among evaluable patients was 67%, including 55% with very good partial response or better and 21% with CR or better, which is comparable to the results observed in the pivotal clinical trial [6].

However, subgroup analyses from the MajesTEC-1 trial revealed that patients with true extramedullary disease (EMD) represent a particularly difficult-to-manage population, demonstrating an ORR of only 35.7% and ≥ CR in 17.9%, which compares unfavorably with other high-risk subgroups [7]. Against this background, we report what to our knowledge is the first reported case of pulmonary parenchymal extramedullary involvement in RRMM successfully treated with teclistamab.

## Case

A 74-year-old man with a medical history of diabetes mellitus and dyslipidemia presented in March 2023 with multiple osteolytic lesions involving the sphenoid sinus, C2 and T8 vertebrae, the ninth rib, left scapula, right sacrum, right pubis, and right femur. He was diagnosed with IgA-κ multiple myeloma. Bone marrow examination revealed ≥ 70% clonal plasma cells. Cytogenetic studies were negative for del(17p), t(4;14), t(14;16), and gain of 1q21. The disease was classified as ISS stage II and R-ISS stage II.

Combination therapy with an anti-CD38 antibody, immunomodulatory drug, and dexamethasone was initiated in April 2023. However, disease progression was noted due to the development of a new sacral lesion. Following local radiotherapy to this lesion, ixazomib, lenalidomide, and dexamethasone were commenced, but serum IgA continued to rise, indicating further progression. In January 2025, isatuximab, carfilzomib, and dexamethasone were administered for three cycles. However, approximately six weeks after achieving a partial response, the left posterior pharyngeal soft-tissue lesion and multiple pulmonary nodules showed rapid progression, with new bilateral hilar lymphadenopathy and a subcutaneous mass in the left upper abdomen. Serum IgA increased by approximately 500 mg/dL during this period. In the absence of microbiological evidence of infection, including negative sputum cultures and β-D-glucan, these findings were considered consistent with disease progression. Bronchoscopic biopsy was not pursued due to the risk of treatment delay and potential deterioration of respiratory status.

At that time, laboratory values were as follows: WBC 4,170/µL, hemoglobin 10.8 g/dL, platelet count 181 × 10⁹/L, LDH 403 U/L, IgG 256 mg/dL, IgA 980 mg/dL, and serum free light chains κ/λ 23.5/0.5 mg/L. The patient received 35 g of intravenous immunoglobulin and one cycle of CHOP chemotherapy. In April 2025, at the age of 76 years with an Eastern Cooperative Oncology Group performance status of 2, treatment with teclistamab was initiated as fifth-line therapy. No notable adverse events occurred following the first step-up dose of 0.06 mg/kg. However, after the second step-up dose of 0.3 mg/kg on day 4, the patient developed grade 1 cytokine release syndrome the following day. As fever did not respond to acetaminophen, tocilizumab was administered, resulting in rapid resolution without recurrence. Immune effector cell–associated neurotoxicity syndrome did not occur during treatment.

**Fig. 1:**
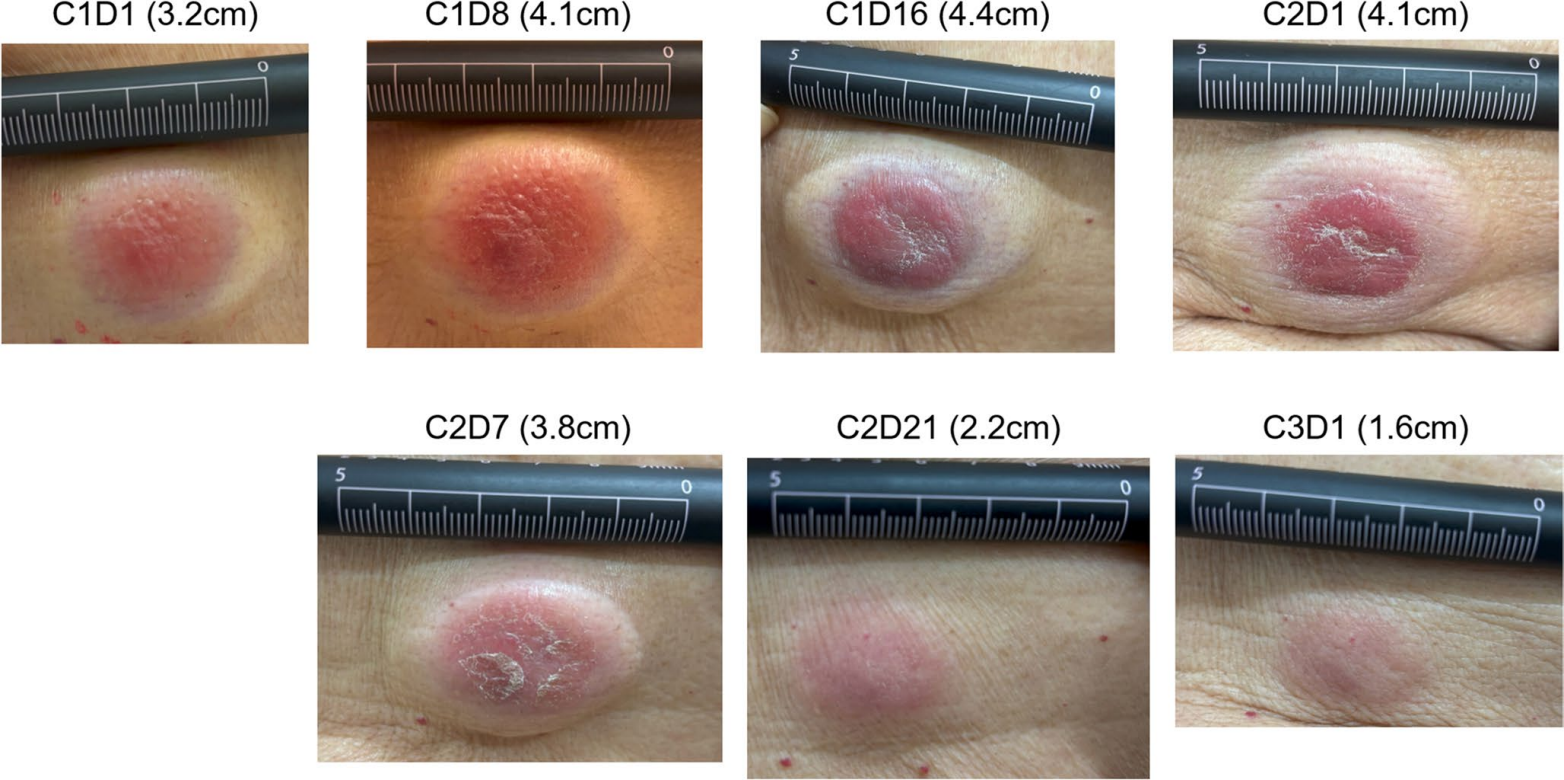
Changes in the abdominal subcutaneous mass after initiation of teclistamab. Subcutaneous mass in the abdominal wall demonstrates dynamic changes after the start of teclistamab therapy. The lesion gradually enlarged during early treatment but subsequently regressed and diminished in size over time, eventually becoming nearly flattened

**Fig. 2:**
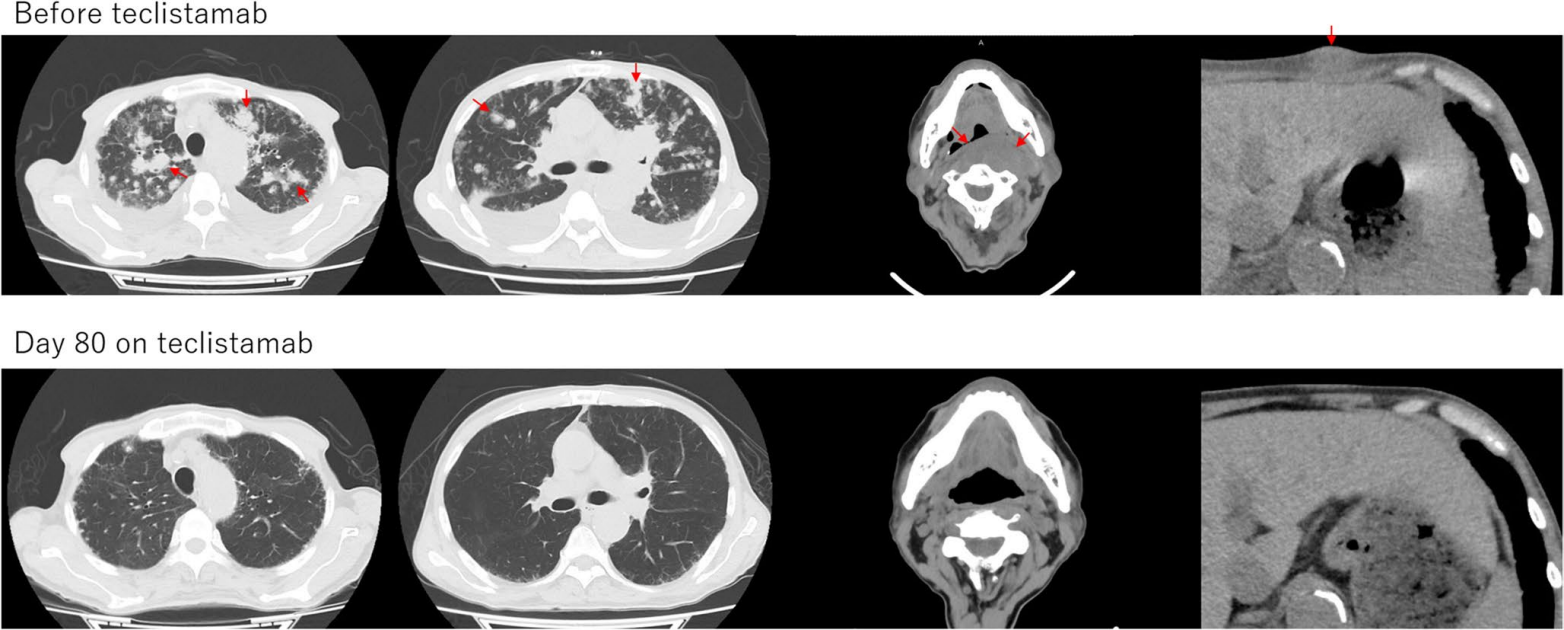
Radiological improvement of pulmonary and soft-tissue extramedullary lesions following teclistamab therapy. Computed tomography images before teclistamab initiation and on day 80 after treatment initiation. Red Arrows indicate multiple pulmonary nodules, the left posterior pharyngeal wall lesion, and the left abdominal subcutaneous mass. Multiple pulmonary nodules and soft-tissue extramedullary lesions evident at baseline showed marked regression, with complete radiologic resolution observed by day 80

A subcutaneous abdominal mass measuring 3.2 cm before teclistamab initiation increased to 4.4 cm on day 16 but subsequently regressed and evolved into a scar by day 52 (Fig. 1). By day 80, all extramedullary lesions, including the pulmonary nodules, had resolved (Fig. 2). At 4.5 months after teclistamab initiation, both positron emission tomography–computed tomography and 10-color multiparameter flow cytometry of the bone marrow demonstrated no detectable minimal residual disease.

**Fig. 3:**
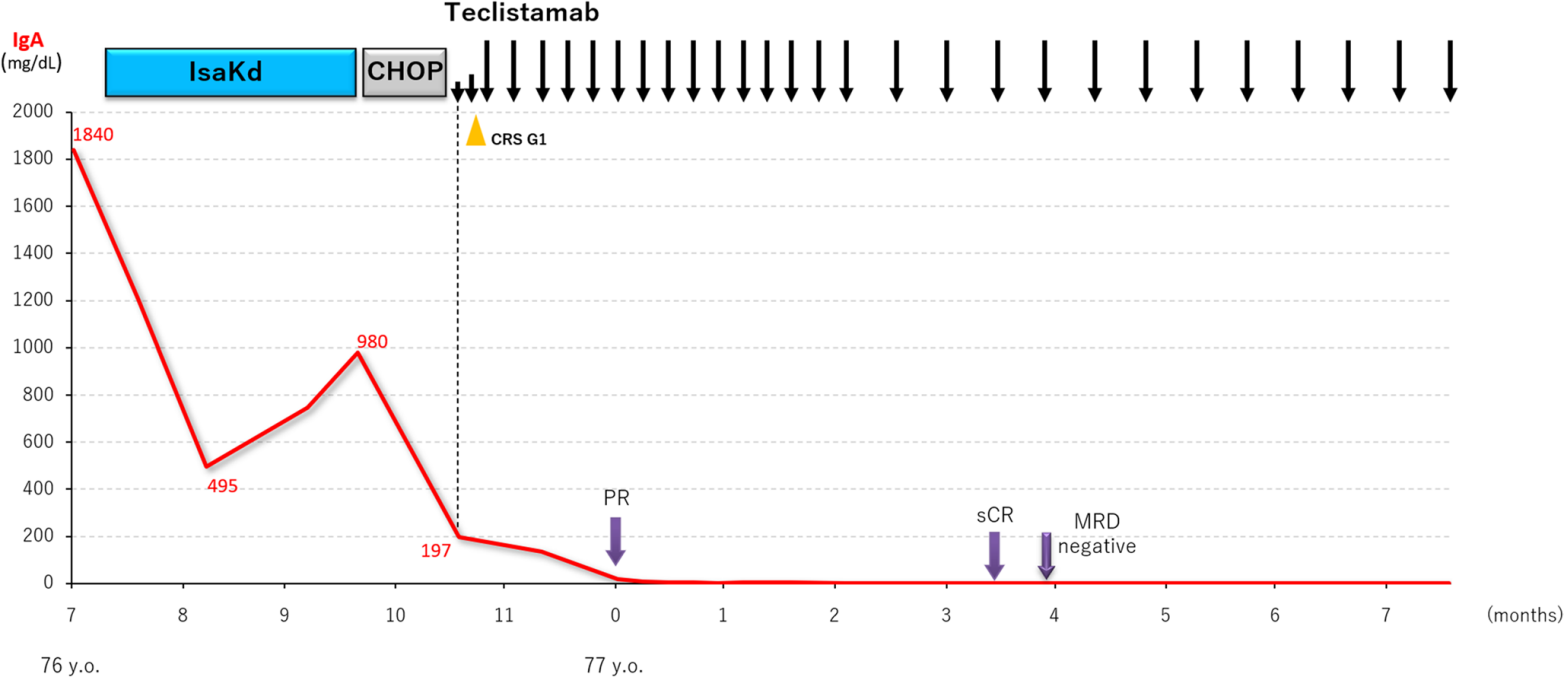
Clinical course and changes in serum IgA levels. Trend of serum IgA levels throughout the treatment course. Despite disease progression during IsaKd, teclistamab following CHOP therapy achieved profound decline in serum IgA levels, with durable disease control thereafter. CRS Grade 1 occurred after the second step-up dose and resolved rapidly. IsaKd, isatuximab, carfilzomib, and dexamethasone; CHOP, cyclophosphamide, doxorubicin, vincristine, and prednisolone; CRS, cytokine release syndrome; PR, partial response; sCR, stringent complete response; MRD, minimal residual disease

During the treatment course, several adverse events were observed, including diffuse erythema involving more than 50% of the anterior chest, back, and extremities on day 66, which resolved promptly following prednisolone 10 mg/day for 5 days. Grade 3 eosinophilia developed on day 80 and also resolved with prednisolone 20 mg/day for 5 days. Reactivation of hepatitis B virus was detected on day 128, and tenofovir therapy was initiated. The patient required a total of 120 g of subcutaneous immunoglobulin replacement over 7 months for hypogammaglobulinemia. At the time of this report, seven months after teclistamab initiation, he continues therapy without evidence of relapse and remains clinically stable (Fig. 3).

## Discussion

Pulmonary extramedullary extramedullary dissemination as the disease progresses, although pulmonary parenchymal involvement is exceedingly rare, occurring in less than 1% of patients [8]. MM with pulmonary involvement has also been reported to carry a dismal prognosis, with an estimated survival of approximately 2.8 to 4 months [9].

We reviewed previously reported cases of MM with pulmonary involvement and summarized them in Table 1 [9–16]. These cases collectively highlight the highly aggressive nature of this condition. For example, a 51-year-old man with light-chain MM developed bilateral hilar lymphadenopathy and multiple pulmonary nodules shortly after allogeneic stem cell transplantation. Bronchoscopic biopsy revealed plasma cell infiltration consistent with recurrent myeloma, and he died of rapidly progressive respiratory failure only 16 days after admission [10]. Another patient in his late sixties, who had previously undergone autologous transplantation and had penta-refractory disease, relapsed with massive pleural effusion and mediastinal shift due to pulmonary extramedullary involvement, clinically manifesting as tension hydrothorax. Despite intensive multi-agent chemotherapy including KD-PACE, his disease remained refractory and he transitioned to palliative care [9]. Similarly, a man in his sixties developed pulmonary disease only one year after diagnosis. Despite treatment with potent novel agents, including venetoclax, carfilzomib, and dexamethasone, his disease did not respond, his performance status declined, and he ultimately elected home hospice care [9]. In contrast, a few reports have described relatively favorable outcomes, including rapid symptomatic improvement and complete resolution of pulmonary masses following introduction of novel agents such as bortezomib [13, 14]. These findings suggest that timely and appropriate therapeutic intervention may occasionally result in meaningful disease control. Nevertheless, pulmonary parenchymal involvement remains a major poor prognostic factor, and it still remains uncertain whether newer immunotherapies targeting BCMA or GPRC5D can substantially improve outcomes in this population.

Extramedullary disease in MM is biologically aggressive and often resistant to conventional therapies. This is partly attributed to plasmablastic morphology or the presence of high-risk cytogenetic abnormalities such as del(17p) or 1q amplification [17]. Such cases are often resistant to standard therapies for MM, including IMiDs, PIs, and anti-CD38 antibodies. Although cytogenetic analysis of the pulmonary lesions was not feasible in our case, the absence of high-risk abnormalities in the bone marrow may have contributed to the favorable response achieved.

Radiographically, pulmonary MM lesions can present with highly variable imaging appearances, making it difficult to distinguish them from disease entities such as lymphoma, post-transplant lymphoproliferative disorder, miliary tuberculosis, metastatic tumors, or infectious pneumonia based on imaging alone [10, 16]. A definitive diagnosis generally requires lung biopsy confirming CD138-positive plasma cells with light-chain restriction. However, in the present case, the coexistence of multiple soft-tissue extramedullary masses along with a rising IgA level strongly suggested clinical progression, leading us to initiate prompt treatment without invasive biopsy. This timely intervention may ultimately have contributed to the successful clinical outcome.

Recently, therapeutic strategies for EMD in MM have evolved with the development of bispecific antibodies. In particular, dual-targeting strategies combining BCMA- and GPRC5D-directed bispecific antibodies have shown promising efficacy, as demonstrated in the RedirecTT-1 study [18] evaluating teclistamab plus talquetamab. In this context, our case highlights that teclistamab may still achieve meaningful responses in selected patients with pulmonary extramedullary involvement.

Pseudoprogression is an important clinical consideration during treatment with immune-based therapies. In our patient, enlargement of the abdominal wall mass was observed shortly after initiation of teclistamab. In lymphoma, pseudoprogression has been documented in approximately 10–20% of patients treated with immune checkpoint inhibitors within the first 12 weeks after therapy initiation [19]. Similarly, pseudoprogression has also been reported in MM during treatment with BCMA-directed bispecific antibodies [20]. The mechanism of pseudoprogression is thought to involve rapid recruitment and accumulation of T cells within tumor lesions, along with transient cytokine-mediated inflammation. Indeed, tumor specimens from patients responding to CAR T-cell therapy have demonstrated increased infiltration of cytotoxic T cells and macrophages. Furthermore, post-treatment elevation of interleukin-8 has been proposed as a potential biomarker associated with pseudoprogression [21]. Given the biochemical improvement in M-protein levels despite apparent tumor enlargement, pseudoprogression was clinically suspected, and treatment with teclistamab was continued, leading to an excellent response. This case suggests that, when treating MM with BsAb therapy, it may be reasonable to continue treatment when clinically feasible, even if transient enlargement of some lesions is observed, provided that the overall clinical situation remains favorable.

This case represents a rare and informative clinical experience demonstrating that teclistamab can provide meaningful therapeutic benefit even in RRMM with extramedullary disease involving the lung, which is generally associated with extremely poor outcomes. Although bispecific antibodies may not always achieve high response rates in RRMM with EMD, given that treatment options for triple-class–exposed RRMM remain limited, the presence of extramedullary involvement alone should not preclude consideration of BsAb therapy. Further accumulation of large-scale real-world data specifically focusing on BsAb therapy in MM patients with pulmonary EMD will be essential to identify the clinical and biological factors that may predict therapeutic benefit.

**Table 1:** Summary of reported cases of multiple myeloma with pulmonary involvement

Year	Age / Sex	Characteristics of pulmonary lesions	Treatment	Outcome	References
2006	51 / M	Bilateral hilar lymphadenopathy and multiple pulmonary nodules	Supportive care for relapse after allogeneic HSCT	Died of progressive respiratory failure 16 days after admission	[10–12]
2007	42 / F	Right anterior mediastinal opacity and multiple pulmonary nodular lesions	Thoracoscopic biopsy (VATS), melphalan + steroids	Symptomatic improvement after 2 months	[11–13]
2010	20 / F	Solid tissue formation within cystic lung lesion	Diagnostic thoracotomy and wedge resection	Excellent prognosis after complete resection	[12, 13]
2010	68 / F	Solid right lower-lobe pulmonary nodule	Diagnostic thoracotomy and wedge resection	Excellent prognosis after complete resection	[12–14]
2012	64 / M	Large apical lung mass (11 × 6 cm)	Bortezomib + dexamethasone	Complete disappearance; stringent CR	[13–15]
2013	62 / F	Multiple bilateral non‑calcified pulmonary nodules	Bortezomib + lenalidomide + dexamethasone	Rapid radiologic resolution; sustained VGPR	[14–16]
2022	65 / M	Heterogeneous pulmonary and pleural masses	Chemotherapy	Lost to follow‑up	[15, 10]
2024	Late 60s / M	Massive pleural effusion with mediastinal shift (tension hydrothorax)	Thoracentesis, selinexor, dexamethasone	No response; progressive respiratory failure; palliation	[9, 10]
2024	Mid‑60s / M	Pulmonary extramedullary plasmacytoma	Venetoclax + carfilzomib + dexamethasone	Clinical deterioration; home hospice	[9, 17]
2026	61 / M	Multiple randomly distributed pulmonary nodules with interstitial thickening	Identified during lenalidomide maintenance	Timely intervention enabled	[16]

*M*, male; *F*, female; *HSCT*, hematopoietic stem cell transplantation; *VATS*, video-assisted thoracoscopic surgery; *CR*, complete response; *VGPR*, very good partial response

## Data Availability

The data supporting the findings of this study are available upon request from the corresponding author. The data were not publicly available because of privacy or ethical restrictions.
